# Prevalence of *Campylobacter* spp. in skinless, boneless retail broiler meat from 2005 through 2011 in Alabama, USA

**DOI:** 10.1186/1471-2180-12-184

**Published:** 2012-08-24

**Authors:** Aretha Williams, Omar A Oyarzabal

**Affiliations:** 1 Department of Biological Sciences, Alabama State University, 1627 Hall Street, Montgomery, AL, USA; 2 IEH Laboratories & Consulting Group, 15300 Bothell Way NE, Lake Forest Park, WA, 98155, USA

## Abstract

**Background:**

The prevalence of *Campylobacter* spp. in 755 skinless, boneless retail broiler meat samples (breast, tenderloins and thighs) collected from food stores in Alabama, USA, from 2005 through 2011 was examined. *Campylobacter* spp. were isolated using enrichment and plate media. Isolates were identified with multiplex PCR assays and typed with pulsed field gel electrophoresis (PFGE). Data were analyzed by nominal variables (brand, plant, product, season, state and store) that may affect the prevalence of these bacteria.

**Results:**

The average prevalence of *Campylobacter* spp. in retail broiler meat for these years was 41%, with no statistical differences in the prevalence by year (*P* > 0.05). Seasons did not affect the prevalence of *C. jejuni* but statistically affected the prevalence of *C. coli* (*P* < 0.05). The prevalence by brand, plant, product, state and store were different (*P* < 0.05). Establishments from two states had the highest prevalence (*P* < 0.05). *C. coli* and *C. jejuni* had an average prevalence of 28% and 66%, respectively. The prevalence of *C. coli* varied by brand, plant, season, state, store and year, while the prevalence of *C. jejuni* varied by brand, product, state and store. Tenderloins had a lower prevalence of *Campylobacter* spp. than breasts and thighs (*P* < 0.05). Although no statistical differences (*P* > 0.05) were observed in the prevalence of *C. jejuni* by season, the lowest prevalence of *C. coli* was recorded from October through March. A large diversity of PFGE profiles was found for *C. jejuni*, with some profiles from the same processing plants reappearing throughout the years.

**Conclusions:**

The prevalence of *Campylobacter* spp. did not change during the seven years of the study; however, it did change when analyzed by brand, product and state. Seasons did not affect the prevalence of *C. jejuni,* but they did affect the prevalence of *C. coli*. Larger PFGE databases are needed to assess the temporal reoccurrence of PFGE profiles to help predict the risk associated with each profile.

## Background

Campylobacteriosis is one of the most important foodborne diseases worldwide. The number of reported cases varies by country. For instance, New Zealand had the highest incidence with 161.1 cases for every 100,000 population in 2008 [1]. Canada had an incidence of 36.1 cases for every 100,000 person per years [2], and European countries have an overall incidence of 48 cases per 100,000 population [3]. In Scotland, there were 95.3 reported cases per 100,000 in 2006 [4]. In the US, campylobacteriosis is the third most important bacterial foodborne disease, with an incidence of 12 cases per 100,000 [5]. *Campylobacter* spp. are still found at high prevalence in retail broiler carcasses [6,7] and in retail broiler meat [8-10]. In the USA, the U. S. Department of Agriculture Food Safety and Inspection Services (USDA FSIS) has recently updated the compliance guideline for poultry slaughter to make the regulations related to *Salmonella* detection more stringent and to enforce the implementation of a performance standard for *Campylobacter* spp. [11].

Although there have been recent reports reviewing the incidence of campylobacteriosis per year [5] and the prevalence of *Campylobacter* spp. in processed carcasses [7], there are no recent reports on the prevalence of these bacteria in retail broiler meat. In addition, the reports of prevalence are always presented without analyzing the data by nominal variables, i.e. processing plant, product, season, state and store that may influence the prevalence of these bacteria in retail broiler meat. This publication summarizes the prevalence of *Campylobacter* spp. in skinless, boneless retail broiler meat from 2005 to 2011. Besides describing the prevalence per year, the prevalence by brand, plant, product, season, state, store, and *Campylobacter* spp. found in the products are described. In addition, the results of typing these *Campylobacter* isolates by pulsed field gel electrophoresis (PFGE) to determine the DNA relatedness of isolates from the same processing plants but from different years are presented.

## Methods

### Sample collection

A total of 755 skinless, boneless retail broiler meat samples were analyzed from 2005 through 2011 from four retail stores in Auburn, Alabama and three stores in Montgomery, Alabama. Samples included breasts, tenderloins (comprised of the muscle pectoralis minor) and thighs. All samples were tray packs of approximately 1–2 lbs. More samples were processed during the months of summer than in winter.

### Sample preparation and enrichment procedures

From each tray pack, 25 g of product were weighed and placed in sterile Whirl-Pak® bags (Nasco, Fort Atkinson, WI). Meat samples were enriched at a 1:4 ratio (w:v) in modified Bolton broth supplemented only with cefoperazone (33 mg per l), amphotericin B (4 mg per l) and 5% lysed horse blood. Samples were enriched for 48 h at 42°C under microaerobic atmosphere (10% CO_2_, 5% O_2_, and 85% N_2_, AirGas South, Inc., Montgomery, AL), which was added to anaerobic jars with an evacuation replacement system (MACSmics Jar Gassing System, Microbiology International, Frederick, MD).

### Isolation of *Campylobacter* spp

Enriched samples (broth) were plated (~0.1 ml) on modified charcoal cefoperazone deoxycholate agar (mCCDA) for isolation and identification of *Campylobacter* spp. In 2009, 2010 and 2011, a slight modification was made to the protocol. For each sample, 0.1 ml of the enrichment broth was transferred to an mCCDA plate using a filter membrane as described elsewhere [12]. All agar plates were incubated at 42°C under microaerobiosis for 48 h. Suspected *Campylobacter* colonies were observed under phase contrast microscopy (Optiphot-2, Nikon Instruments Inc., Melville, NY, or BX51, Olympus America Inc., Center Valley, PA) for their spiral morphology and darting motility. A small amount of growth from each plate was transferred to modified Campy-Cefex (mCC) agar plates supplemented with cefoperazone (33 mg), amphotericin B (4 mg) and 5% lysed horse blood. Plates were incubated at 42°C for 24 h under microaerobic conditions, and from these plates DNA was extracted using the Wizard® Genomic DNA Purification Kit as described by the manufacturer (Promega, Madison, WI) but without the RNA digestion step, and plugs were made for PFGE analysis. Isolates were stored at −80°C in tryptic soy broth (TSB, Difco, Detroit, MI) supplemented with 30% glycerol (vol/vol) and 5% horse blood.

### Identification of isolates using mPCR assays

Isolates were identified as *C. jejuni* or *C. coli* using two multiplex PCR (mPCR) assays: one based on primers targeting the *ask* gene of *C. coli*[13] and the *hipO* gene of *C. jejuni*[14], and the other targeting the *ask* gene of *C. coli* (different primers from the previous mPCR) and the *glyA* gene of *C. jejuni*[15]. PCR assays were performed in 25 μl aliquots using pre-made mixes of GoTaq® (Promega) or EconoTaq® PLUS (Lucigen, Middleton, WI). The assays were performed in a DNA Engine® Thermal Cycler (Bio-Rad Laboratories, Hercules, CA) as previously described [10,15]. Amplified products were detected by gel electrophoresis stained with ethidium bromide and visualized using the VersaDoc™ Imaging System (Bio-Rad Laboratories).

### PFGE analysis of isolates

PFGE was performed on isolates as previously described [15,16]. Briefly, DNA was digested with *Sma*I and separated using a CHEF DR II system (Bio-Rad Laboratories). *Salmonella enterica* subsp*. enterica* serovar Braenderup strain H9812 (ATCC BAA-664) was used as the DNA size marker, and TIFF images of gels stained with ethidium bromide were loaded into BioNumerics version 6 (Applied Maths, Austin, TX) for analysis. Pairwise-comparisons were performed with the Dice correlation coefficient, and cluster analyses were performed with the unweighted pair group mathematical average (UPGMA) clustering algorithm. The optimization and position tolerance for band analysis were set at 2 and 4%, respectively, and similarity among PFGE restriction patterns was set at 90% [17].

### Diversity index calculation

To assess the diversity of the PFGE profiles, the SID was calculated for the PFGE grouping and by *Campylobacter* spp. (*C. jejuni* or *C. coli*) [18,19].

### Statistical analysis

Results were analyzed with the Fisher's Exact Test for count data and the Kruskal-Wallis test to determine differences in nominal variables (brand, plant, product, season, state and store). The confidence interval (95%) for each proportion of positive per year was also calculated. Statistical differences were set at P ≤ 0.05 and P ≤ 0.01 for the chi-square and the Kruskal-Wallis tests, respectively. Data were not assumed to have a normal distribution. All the statistical analyses were performed with R [20].

## Results

From 755 samples analyzed, 308 (41%) were positive for *Campylobacter* spp., with 85 (28%) of the isolates identified as *C. coli* and 204 (66%) identified as *C. jejuni*. Nineteen isolates (6%) were presumptively identified as *Campylobacter* spp. but were not recoverable from −80°C. These isolates were lost between 2005 and 2009 (Tables 1 and 2). The average prevalence of *Campylobacter* spp. in retail broiler meat per year had a standard deviation of 5.4, and the standard deviation for the average prevalence for *C. coli* and *C. jejuni* was 18 and 17, respectively. Table 1 shows the prevalence of *Campylobactercoli* and *C. jejuni* per year.

**Table 1:** **Number of samples tested by year and prevalence of *C. coli *and *C. jejuni *in retail meat products, 2005 through 2011**

**Year**	**No. Samples**	**% Positive**^a^	***C. jejuni *(%)**	***C. coli *(%)**
2005	92	47	14 (33)	28 (65)
2006	87	34	22 (73)	6 (20)
2007	148	45	40 (60)	24 (36)
2008	131	40	36 (68)	10 (19)
2009	72	46	21 (64)	6 (18)
2010	109	39	37 (86)	6 (14)
2011	116	34	34 (87)	5 (13)

^a^ Isolates lost by year: 2005 = 1; 2006 = 2; 2007 = 3; 2008 = 7 and 2009 = 6

No statistical difference was found for the number of positive samples from 2005 through 2011 (Fisher’s exact test for the difference 2005 vs. 2011: p = 0.063).

**Table 2:** ***Campylobacter *spp. from retail broiler samples identified by multiplex PCR assays**

**Product**	**No. Samples**	**Positive (%)**	***C. jejuni *(%)**	***C. coli *(%)**	**Lost Isolates (%)**
Breasts	302	119 (39)	78 (66)	37 (31)	4 (3)
Tenderloins	195	51 (26) ^a^	29 (57)	17 (33)	5 (10)
Thighs	258	138 (53)	97 (70)	31 (22)	10 (7)
Total	755	308 (41)	204 (66)	85 (28)	19 (6)

^a^ Fisher's exact test: breasts vs. tenderloins: p = 0.003; thighs vs. tenderloins: p = <0.001.

Table 2 shows the percentage distribution of *C. coli* and *C. jejuni* by product (breast, tenderloin or thigh). The Fisher's Exact Test for count data showed that tenderloins had a lower prevalence of *Campylobacter* spp. than breasts (*P* = 0.003) and thighs (*P* < 0.001). In 2005, the ratio *C. coli*:*C. jejuni* was different from the other years, with a higher percentage of *C. coli* than *C. jejuni* for that particular year (Table 1).

No statistical differences were seen in the prevalence of *C. jejuni* by season (Table 3 and Table 4), although the months of October through March showed the highest number of *C. jejuni* and the lowest number of *C. coli* (Table 3). The data showed that two states had processing plants where the prevalence was highest (Table 5), and the Kruskal-Wallis (KW) rank sum test for categorical variables showed again that the prevalence of *C. jejuni* was not influenced by season. However, the prevalence was influenced by brand, plant, product, state and store (Table 4). The prevalence of *C. coli* appeared to vary by brand, plant, season, state and store.

**Table 3:** **Prevalence of *Campylobacter *spp. by season. J-M: January-March; A-J: April-June; JY-S: July-September; O-D: October-December**

			**Percentage**	
**Months**	**No-samples**	**Positive (%, UCI-LCI**^a^**)**	***C. jejuni***	***C. coli***
J-M	124	50 (40, 49–31)	88	10
A-J	285	116 (41, 46–34)	66	30
JY-S	311	131 (41, 47–36)	56	34
O-D	35	11 (34, 49–17)	91	9

^a^ Upper and lower confidence intervals.

No statistical difference was found for the number of positives by season.

**Table 4:** **Kruskal-Wallis (KW) rank sum test results for the analysis of the prevalence of *Campylobacter *spp. (*C. coli *and *C. jejuni*) by brand, plant, product, season, state and store**

**Nominal variables**	***Campylobacter *spp.**		**P value**	
	**KW Test**	**P value**	***C. coli***	***C. jejuni***
Brand	30.52	<0.001	<0.001	0.006
Plant	43.98	<0.001	<0.001	0.124
Product	33.33	<0.001	0.596	<0.001
Season	1.64	0.649	0.034	0.068
State	34.08	<0.001	<0.001	0.014
Store	18.11	<0.001	<0.001	0.008
Year	7.34	0.289	<0.001	0.196

**Table 5:** **Prevalence of *Campylobacter *spp. by state and processing plant. The processing plants from GA and MS had the highest prevalence (*P*  < 0.05)**

**State**	**Processing plant (Number of samples)**^a^	**Positive (%)**
GA	B (121)	47.9
	I (29)	48.3
	J (53)	58.5
	R (51)	43.1
MS	D (10)	44.4
	O (193)	49.5
NC	E (27)	40.7
	H (116)	25.0
	N (72)	36.1
TN	L (24)	33.3
TX	Q (23)	30.4
VA	M (17)	11.8

^a^ Plants from GA and MS = 456 samples; Plants from NC, TN, TX and VA = 279 samples. Plants A, C, F, G, K and P each represented less than 10 samples.

**Figure 1:**
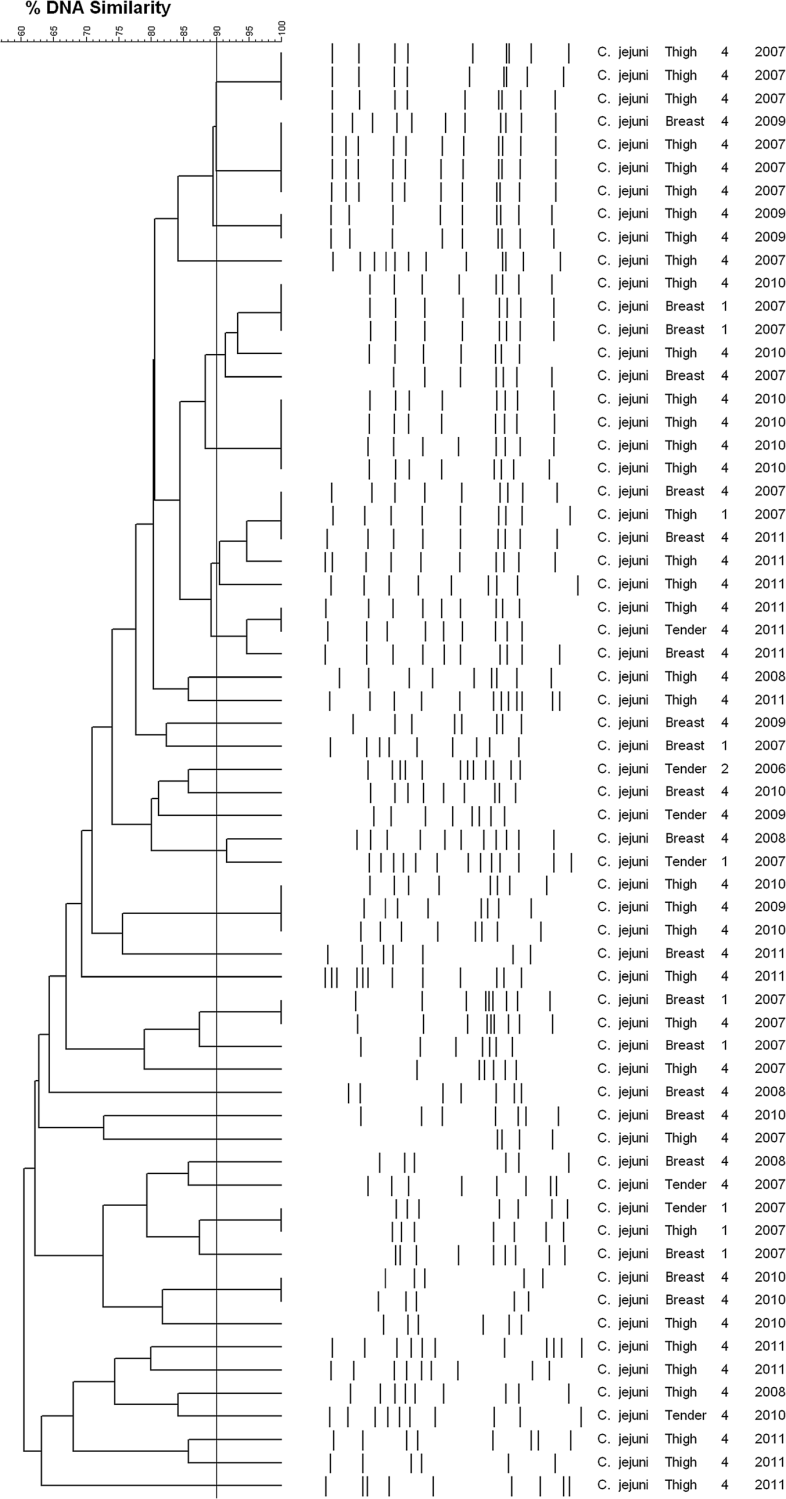
**Diversity of PFGE profiles.** This picture shows the diversity of the *C. jejuni* PFGE profiles from the same processing plant but different years. PFGE patterns re-appeared at different years, suggesting that few predominant PFGE patters are associated to a given processing plant. A cut-off of 90%, based on previous studies [32,36], was used to separate PFGE subtypes.

PFGE analysis of isolates from the same processing plants but from different years showed a large variability of PFGE profiles. However, some PFGE types re-appeared in different years (Figure 1). Table 6 shows the Simpson's index of diversity (SID) for 175 *C. jejuni* isolates and 78 *C. coli* isolates, including the variability of types by years for *C. jejuni* isolates.

**Table 6:** **Comparison of the Simpson’s index of diversity (SID) between *C. jejuni *and *C. coli***

**Species**	**Number of unrelated strains**	**Number of types**	**SID**
*C. coli*	78	24	0.924
*C. jejuni*	175	87	0.982
*C. jejuni* by year			
2005	15	14	0.989
2006	19	11	0.918
2007	39	22	0.950
2008	23	20	0.988
2009	15	11	0952
2010	31	20	0.959
2011	33	25	0.979

## Discussion

There have not been recent reports on the prevalence of *Campylobacter* in retail broiler meat in the USA. Most of the studies include products with skin, and the samples are taken during processing where the carcasses are still intact and before portioning. The more recent publication summarizing the prevalence of *Campylobacter* spp. in processed carcasses comes from the nationwide microbiological baseline data collection program by the USDA FSIS. These data were collected from July 2007 through June 2008 and showed a prevalence of 40% *Campylobacter* positive in carcasses post-chill [7]. Yet, most of the broiler meat sold in stores across the US is sold in tray packs and include boneless, skinless products.

Because *Campylobacter* spp. are at low numbers in retail broiler meat in the USA [7], concentration by centrifugation [21] and filtration have been performed to increase the number of *Campylobacter* cells before plating [8,22]. Bolton broth was used in this study because this medium has been used most frequently for isolation of *Campylobacter* from poultry samples [23,24], and it appears to be one of the best available alternatives to compromise between the inhibition of competitors and the growth of *Campylobacter* spp. [25]. The data in Table 1 are similar to most recent reports on the prevalence of *Campylobacter* spp. in retail samples in the US [9,10,21]. This prevalence is similar to the data from Belgium [26], but lower than the reports from Ireland [27], England [28,29], Canada [30], Japan [31] and Spain [32]. The prevalence among different countries varies from as low as 25% in Switzerland to as high as 100% in the Czech Republic [31,33,34].

The low prevalence of *Campylobacter* spp. in tenderloins has been previously reported [9,10]. The fluctuation in the prevalence of *C. coli* and *C. jejuni* by year has not been previously addressed. However, more surveillance data is necessary to understand the extent of this fluctuation, which may be comprised of an actual variability by year and/or an artifactual variability due to the methodology used for isolation. It has been shown that analyzing more than 25 g of sample increases the chances of recovering positive samples for *Campylobacter* spp. [35]. Therefore, there is no optimal methodology to determine the true prevalence of these bacteria, and for all purposes the actual prevalence of *Campylobacter* spp. in retail broiler meat may be underestimated. An optimal methodology that could detect the true number of positive samples and/or the samples with the highest number of *Campylobacter* spp. would provide a more accurate prevalence for surveillance purposes of these pathogens in retail broiler meat.

There is substantial information suggesting that the predominant *Campylobacter* spp. present in commercial broiler products are *C. jejuni* and *C.coli*, a trend that is especially clear in industrialized nations [27,28,36]. Because *Campylobacter* spp. are inert, very few biochemical tests are used for identification of species. These tests are mainly performed in qualified laboratories studying the taxonomy of these bacteria where several controls are evaluated in parallel to avoid false identification. Therefore, molecular techniques, mainly the polymerase chain reaction validated by sequencing and Southern blotting, provide simple, robust identification to the species level. In a recent summary of the current *Campylobacter* spp. worldwide prevalence, *C. jejuni* was the predominant *Campylobacter* spp. isolated from retail poultry with the exception of Thailand and South Africa, where the predominant species was *C. coli*[31]. In some countries, *C. coli* represents less than 20% of all the *Campylobacter* isolates found in retail broiler meats [31,37,38]; yet, they are at a prevalence that exceeds 20% in live broiler chickens. This difference may be explained by the isolation procedure: direct plating is used to analyze fecal material from live animals, while enrichment is used to analyze retail broiler meat. Both *Campylobacter* spp. have been found in enriched retail samples [10], but it is not clear if enrichment procedures hinder one species versus the other, or favor the species that contain more vegetative cells at the beginning of the enrichment procedure.

Although other countries, such as Denmark, have shown a strong seasonal correlation in the prevalence of *Campylobacter* spp. in broiler flocks and in retail broiler meat [38], there were no seasonal variations detected in *C. jejuni*. Although statistical differences were seen for *C. coli*, a larger database is needed to confirm these results. There is no long-term data to assess the changes in the prevalence of *Campylobacter* spp. present in retail broiler meats. The results from 2005 clearly show that *C. coli* was the predominant species. These strains were tested with the same PCR assays as the rest of the data set; therefore, there is no bias in the methodology for identification. These data suggest that the product, the processing plant, the region, and even the season, may impact the prevalence of these pathogens in retail broiler meats.

A large diversity in the PFGE profiles of *Campylobacter* spp. has been reported in the literature [8,28], with the greatest diversity found in *Campylobacter* isolates from broiler chickens [28]. This diversity can be related to the larger database available for broiler chickens. This diversity may also be due to a true variability of types, meaning that *Campylobacter* strains found in chickens show more diversity than the *Campylobacter* strains isolated from other animal species. The diversity of *Campylobacter* strains by PFGE has also been demonstrated in clinical samples. For instance, throughout an infection involving 52 patients, one patient had two different *Campylobacter* species and four patients had different *Campylobacter* strains based on PFGE analysis. Although human infections with more than one *Campylobacter* strain are rare, changes in the PFGE profiles throughout an infection complicates the epidemiological studies of *Campylobacter* spp. [39]. The collection and analysis of retail samples immediately before consumer exposure is the most appropriate sampling point for the collection of data that can be factored into risk analysis models. Therefore, a PFGE database of retail isolates that could be compared to PFGE patterns from human isolates may provide invaluable information to assess the actual risk of humans acquiring campylobacteriosis via consumption of retail meats.

## Conclusions

The prevalence of *Campylobacter* spp. has not changed in the last seven years, and there is no variation in the prevalence due to seasons for *C. jejuni*. However, a seasonal prevalence was found for *C. coli*. Two states yielded more positive samples than four other states. The predominant species was *C. jejuni*, and PFGE analyses indicated a large diversity of types throughout the years. Some of the same PFGE types reoccurred from year to year within samples from the same processing plant. A continuous surveillance of *Campylobacter* spp. in retail broiler meat will provide larger PFGE databases to better assess the reoccurrence of PFGE profiles on a spatial and temporal fashion.

## Competing interests

The authors declare that no competing interests exist.

## Authors' contributions

AW collected and analyzed part of the samples and identified the isolates. AW performed the PFGE analysis. OAO conceived and coordinated the study and designed and revised the manuscript. All authors read and accepted the final version of the manuscript.
